# Vancomycin Treatment of Infective Endocarditis Is Linked with Recently Acquired Obesity

**DOI:** 10.1371/journal.pone.0009074

**Published:** 2010-02-10

**Authors:** Franck Thuny, Hervé Richet, Jean-Paul Casalta, Emmanouil Angelakis, Gilbert Habib, Didier Raoult

**Affiliations:** 1 Unité de Recherche sur les Maladies Infectieuses et Tropicales Emergentes, Université de la Méditerranée, Marseille, France; 2 Service de Cardiologie, Hôpital de la Timone, Marseille, France; 3 Pôle de Maladies Infectieuses, Hôpital de la Timone, Marseille, France; Charité-Universitätsmedizin Berlin, Germany

## Abstract

**Background:**

Gut microbiota play a major role in digestion and energy conversion of nutrients. Antibiotics, such as avoparcin (a vancomycin analogue), and probiotics, such as *Lactobacillus* species, have been used to increase weight in farm animals. We tested the effect of antibiotics given for infective endocarditis (IE) on weight gain (WG).

**Methodology/Principal Findings:**

Forty-eight adults with a definite diagnosis of bacterial IE (antibiotic group) were compared with forty-eight age-matched controls without IE. Their body mass index (BMI) was collected at one month before the first symptoms and one year after hospital discharge. The BMI increased significantly and strongly in vancomycin-plus-gentamycin–treated patients (mean [±SE] kg/m^2^, +2.3 [0.9], *p* = 0.03), but not in controls or in patients treated with other antibiotics. Seventeen patients had a BMI increase of ≥10%, and five of the antibiotic group developed obesity. The treatment by vancomycin-plus-gentamycin was an independent predictor of BMI increase of ≥10% (adjusted OR, 6.7; 95% CI, 1.37–33.0; *p* = 0.02), but not treatment with other antibiotics. Weight gain was particularly high in male patients older than 65 who did not undergo cardiac surgery. Indeed, all three vancomycin-treated patients with these characteristics developed obesity.

**Conclusions/Significance:**

A major and significant weight gain can occur after a six-week intravenous treatment by vancomycin plus gentamycin for IE with a risk of obesity, especially in males older than 65 who have not undergone surgery. We speculate on the role of the gut colonization by *Lactobacillus* sp, a microorganism intrinsically resistant to vancomycin, used as a growth promoter in animals, and found at a high concentration in the feces of obese patients. Thus, nutritional programs and weight follow-up should be utilized in patients under such treatment.

## Introduction

The prevalence of obesity is a major world health problem that is rapidly increasing in developing countries [1], [2]. Currently, more than half of the US population is overweight [3], [4] and thus exposed to a high cardiovascular risk. Obesity results from a mixture of genetic background and environmental factors, including food availability, social networks [5], high fat diet, and physical inactivity [6], [7]. Recent evidence suggests that gut microbiota play a major role in the digestion an energy conversion of nutrients [8], [9].

Moreover, metagenomics studies have demonstrated that the gut microbiota composition may be altered by environmental modifications [10] and differs in lean vs. obese animals and humans [11], [12], suggesting its potential role in the pathogenesis of obesity [13], [14]. A recent 16S rRNA sequencing-based investigation showed that *Firmicutes* are increased in obese mammals [15]. Additionally, a large body of experimental evidence and empirical data from the food industry has shown that *Firmicute* probiotics (*e.g.*, *Lactobacillus* and *Enterococcus*) and antibiotics that modify the gut microbiota can act as growth promoters, increasing the size and weight of animals [16], [17], [18]. Moreover, we recently found that *Lactobacillus* was significantly increased in the stools of obese patients [19]. Avoparcin, an analogue of vancomycin, has been largely used in farm animals [20] and is active on most *Firmicutes*, with the notable exception of *Lactobacillus* species.

Similar growth effects have been observed in children with the long-term use of tetracycline [21], and a possible link between increased antibiotic use and obesity has been proposed [22]. The mechanisms by which antibiotics improve growth performance are not well known, and the selection of gut microorganisms with potential growth promoting effects is one of hypotheses [14], [23]. *Lactobacillus* species are thought to be growth promoters, with an intrinsic vancomycin-resistance [24], [25] and a probiotic activity [26], [27]. Their presence has been shown to dramatically increase food conversion and weight increase in chickens [28], and some reports suggest similar effects in children [29].

Taking advantage of a large cohort of patients treated for infective endocarditis (IE) in our center over many years, we sought to determine the impact of antibiotic therapy, given during a long period at high doses levels, on body weight changes. We retrospectively compared the body mass index (BMI) changes of patients one month before the occurrence of the first symptoms compatible with IE and one year after hospital discharge for a group of patients treated for definite IE and a control group.

## Methods

### Ethics Statement

Written informed consent was obtained from all participating patients and approved by the institutional review board (Comité d'Ethique de l'IFR 48) under reference 08–002.

### Patients

We conducted a retrospective analysis in consecutive adults referred for a suspicion of IE to the Department of Cardiology of La Timone Hospital, Marseille, France, from January 2002 to December 2007. A structured, standardized questionnaire was used to collect demographic, clinical, biological, microbiological, echocardiographic, and therapeutic data in all of these patients. The modified Duke criteria [30] were applied to all suspected cases, which were characterized as definite, probable, or excluded. The weight, height, and BMI (weight [Kg]/height^2^ [m^2^]) of each patient were collected at two different times, one month before the first symptoms (baseline) and one year after discharge, during systematic and standardized consultations or phone contacts. Finally, we included two groups of patients. The “Antibiotic group” was formed by patients who were definitely diagnosed with bacterial IE and treated by intravenous antibiotics for at least four weeks. We defined the control group (same sample size) as consecutives patients first suspected to have IE but finally receiving an excluded diagnosis. Controls did not receive antibiotics for more than fifteen days. The exclusion criteria were: the absence of weight or height data at baseline or at one year, all conditions (IE or others) responsible for a major limitation of physical activity, and changes of weight related to edema or ascites.

### Antibiotic Therapy

The patients in the Antibiotic group received antibiotic therapy according to a standardized local protocol [31]. The patients were stratified according to the type of antibiotics used in association with gentamycin, namely vancomycin, amoxicillin, or other antibiotics (including patients treated with oxacillin and those who alternatively received amoxicillin and vancomycin). The duration of antibiotic therapy ranged from four to six weeks. For each case of IE, the decision of valvular surgery was taken in a multidisciplinary way. In controls with a non-infective valvular heart disease, the indications of cardiac surgery were based on international recommendations [32].

### Questionnaire of Lifestyle

A standardized questionnaire about their life habits was retrospectively proposed within one year after hospital discharge to all of the patients in the two groups. This questionnaire included the following questions: did you quit smoke? (yes/no); were you on a hypocaloric diet because of a weight gain? (yes/no); did you consume probiotics? (yes/no); and did you increase or decrease your physical activity? (increase, decrease, or no change).

### Statistical Analysis

The baseline BMI was that obtained one month before the first symptoms. The changes of BMI at one year were compared between the two groups, and we tested the potential role of the different antibiotics used. Proportions were compared using the Chi-squared test or the Fisher's exact test. For the continuous variables, the Wilcoxon test and Mann-Whitney test were used for comparison between the two groups. Logistic regression was used to determine the predictors of an increase of BMI ≥10% at one year. The following variables were tested as potential predictors: age, sex, cancer, chronic renal insufficiency, the Charlson comorbidity index [33], stroke, heart failure, cardiac surgery within the year after admission, and the type of antibiotic used in association with gentamycin (*i.e.*, vancomycin, amoxicillin, or other antibiotics). *P*<0.05 was considered significant, and a Bonferroni's correction was used in case of multiple comparisons. All statistical analyses were performed using EpiInfo software version 3.4.1 (Centers for Disease Control and Prevention, Atlanta, GA, USA).

## Results

### Patient Characteristics

During the period of study, 2,533 patients were referred for a suspicion of IE. Among them, 48 patients with definite IE fulfilled the inclusion criteria, had no exclusion criterion, and were thus included in the “Antibiotic group”. The control group was then formed by another 48 consecutive patients that received an excluded diagnosis of IE and did not meet the study's exclusion criterion.

The clinical characteristics of the two groups are summarized in the Tables 1 and 2. The weight and BMI one month before the first symptoms (baseline) did not differ significantly between the two groups (Antibiotic vs. Control group; weight [Kg], 75.2±17 vs. 69 .8±15, *p* = 0.10; BMI [Kg/m^2^], 25.3±5 vs. 23.9±4, *p* = 0.14). No significant difference was observed between the two groups in terms of age, sex, diabetes, chronic renal insufficiency, comorbidity index, previous heart disease, presence of prosthetic valve, history of heart failure, or smoking behavior. However, a history of cancer was more frequently noticed in the Antibiotic group (20.8% vs. 4.2%, *p* = 0.03), and a cardiac surgery within one year after admission was also performed more frequently in the Antibiotic group (72.9% vs. 16.7%, *p*<0.0001).

**Table 1:** Clinical characteristics of patients in the Antibiotic and control groups.

	Antibiotic group	Controls	*p*
	n = 48	n = 48	value
Age, mean ±SD, y	59.5±14	63.2±14	0.20
Male	36 (75)	29 (60.4)	0.13
Baseline* weight, mean ±SD, Kg	75.2±17	69.8±15	0.10
Baseline* BMI, mean ±SD, Kg/m^2^	25.3±5	23.9±4	0.14
Diabetes	2 (4.2)	1 (2.1)	1.0
Cancer	10 (20.8)	2 (4.2)	0.03
Chronic renal insufficiency	3 (6.3)	1 (2.1)	0.62
Previous heart disease	33 (68.8)	37 (77.1)	0.36
Prosthetic valve	14 (29.2)	18 (37.5)	0.39
Pacemaker, implantable cardioverter defibrillator	11 (22.9)	5 (10.4)	0.10
Stroke	5 (10.4)	0 (0)	0.06
Heart failure	19 (39.6)	19 (39.6)	1.0
Quit smoking†	1 (2.5)	1 (2.5)	1.0
Cardiac surgery with the year after admission	35 (72.9)	8 (16.7)	<0.0001

Values are number (%).

BMI = body mass index.

*Baseline is defined as one month before the first symptoms.

† Defined as yes if smokers reported that they had quit smoking since the previous period. Data available in 40 patients.

**Table 2:** Causes of hospitalization in the 48 controls.

	n
Unexplained fever in patients with heart valve disease or PPM or ICD	15
Fever related to an acute degenerative mitral chordae rupture	7
Respiratory tract infection	7
Pericarditis, myocarditis	4
Paraprosthetic leak without IE	5
PPM or ICD local pocket infection	2
Local catheter related infection in patientwith heart valve disease	2
Stroke	1
Chronic lymphoid leukemia	1
Urinary tract infection in patientwith heart valve disease	1
Systemic lupus	1
Fever related to neuroleptic treatment	1
Myxoma	1

PPM = pacemaker; ICD = implantable cardioverter defibrillator.

In the Antibiotic group, 28 patients received amoxicillin for four to six weeks in association with gentamycin for six, four, three, or two weeks in five, three, three, or 17 patients, respectively. Eleven patients received vancomycin for four to six weeks in association with gentamycin for six, two, or one weeks in six, three, or eight patients, respectively. Nine patients received other antibiotics.

### BMI Changes

**Figure 1:**
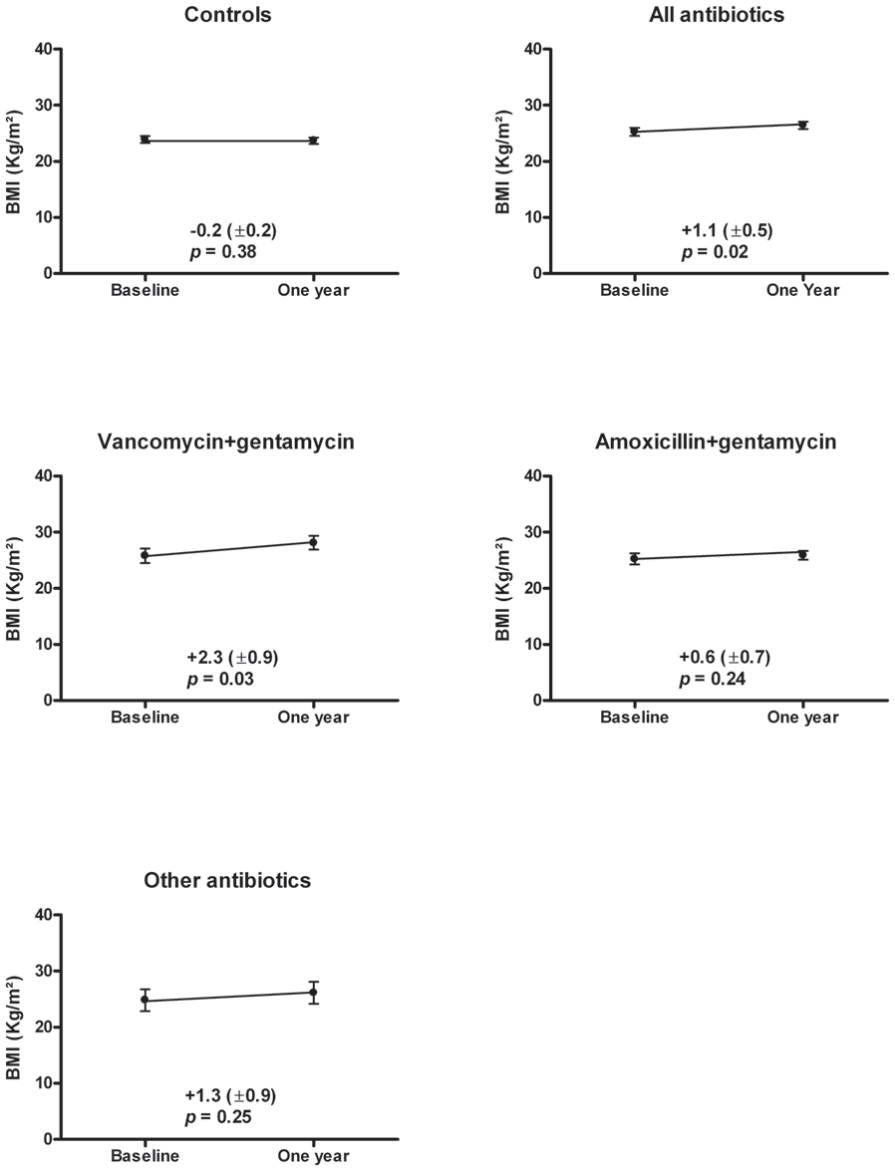
Body mass index (BMI) changes in controls and in patients according to their antibiotic treatment.

The BMI increased significantly in the Antibiotic group (mean [±SE] Kg/m^2^, +1.1 [0.5], *p* = 0.02) but not in the controls (mean [±SE] Kg/m^2^, −0.2 [0.2], *p* = 0.38). After stratification according to the type of antibiotics, BMI significantly and strongly increased only after treatment by vancomycin+gentamycin (mean [±SE] Kg/m^2^, +2.3 [0.9], *p* = 0.03) (Figure 1). No significant BMI increase was observed with amoxicillin+gentamycin or the other antibiotics.

**Figure 2:**
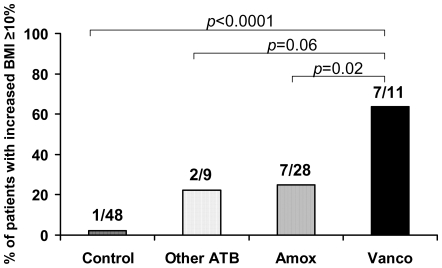
Percentage of patients with a major increase (≥10%) of body mass index (BMI), defined as an increase.

Seventeen patients had a BMI increase of ≥10%: 16 (33.3%) in the Antibiotic group and only one (2.1%) in the control group (*p*<0.0001). This major increase of BMI was observed at a rate of 63.4% after treatment with vancomycin+gentamycin, 25% after treatment with amoxicillin+gentamycin, and 22.2% after treatment with other antibiotics (*p*<0.0001). The rate of BMI increase ≥10% was significantly higher after vancomycin+gentamycin treatment than in controls (*p*<0.0001) or after a treatment by amoxicillin+gentamycin (*p* = 0.02) or other antibiotics (*p* = 0.06) (Figure 2). Among all 96 patients assessed, five developed obesity, defined as a new BMI >30 Kg/m^2^ after an increase of their BMI ≥10%. Three of those had been treated with vancomycin+gentamycin and two with amoxicillin+gentamycin.

After multivariable analysis, treatment with vancomycin+gentamycin remained the only predictor of a major increase of BMI (adjusted OR, 6.7; 95% CI, 1.37–33.0; *p* = 0.02) (Table 3).

**Table 3:** Predictors of an increase of BMI ≥10%.

		Univariate analysis			Multivariable analysis	
	OR	95% CI	*p*	Adjusted OR	95% CI	*p*
Age	1.0	0.96–1.04	0.88			
Male sex	3.2	0.61–16.7	0.17			
Cancer	0.8	0.18–3.73	0.80			
Chronic renal insufficiency	1.0	0.08–11.9	1.0			
Comorbidity index ≥2	1.3	0.38–4.65	0.67			
Stroke	0.6	0.06–5.58	0.62			
Heart failure	0.2	0.23–0.06	0.05	0.2	0.04–1.0	0.05
Cardiac surgery	0.7	0.20–2.76	0.65			
Vancomycin+gentamycin	5.4	1.29–23.0	0.02	6.7	1.37–33.0	0.02
Other antibiotics	2.1	0.27–16.8	0.47			
Amoxicillin+gentamycin	0.4	0.12–1.39	0.15			

**Figure 3:**
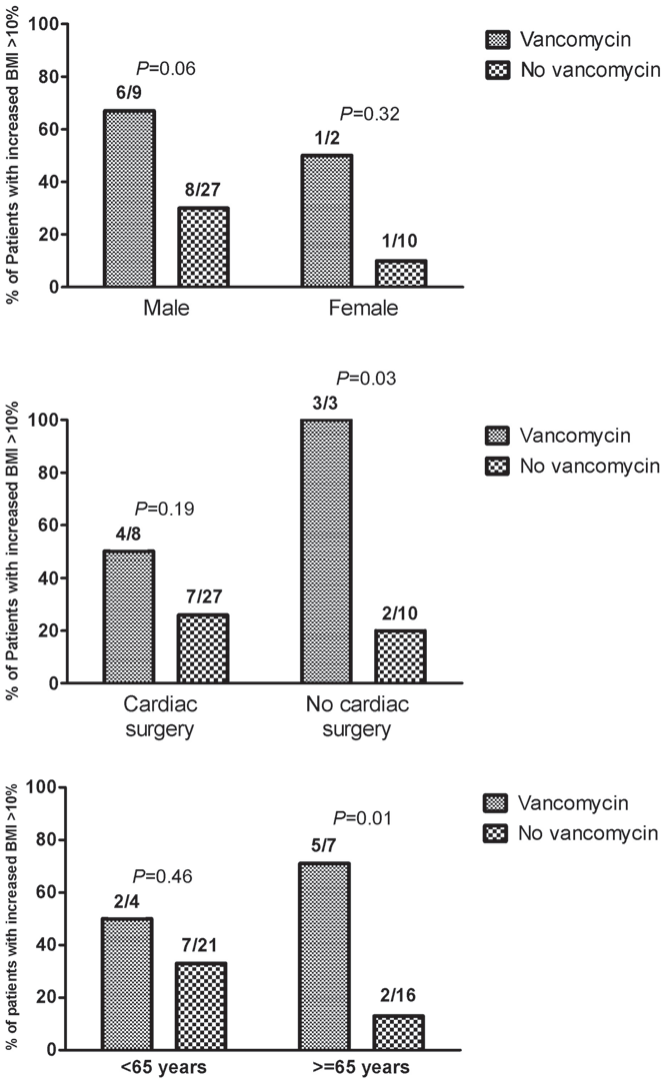
Impact of vancomycin treatment on the risk of major increase of body mass index (BMI ≥10%) in patients treated for infective endocarditis according to age, gender, and cardiac surgery.

By stratified analysis, the impact of vancomycin on the risk of increased BMI ≥10% was particularly high in males (*p* = 0.06), in patients older than 65 (*p* = 0.01), and in the absence of cardiac surgery (*p* = 0.03) (Figure 3). Three patients in the study were males, older than 65, and underwent vancomycin treatment without surgery. All of them became obese after a BMI increase ≥10%, with a weight increase of 20 Kg, 17 Kg, and 10 Kg, respectively.

No significant difference in lifestyle was noticed according the different groups.

## Discussion

The main result of the present work shows a significant and persistent weight gain after an episode of IE in patients who had been treated by vancomycin and gentamycin.

Antibiotics promote weight gain and were among the first growth promoters used in agriculture. Indeed, avoparcin, a glycopeptide structurally related to vancomycin, was widely used in Europe as a growth promoter from the early 1970s until a recent ban due to the emergence of vancomycin-resistant enterococci [20]. This antibiotic has been shown to improve feed efficiency and increases weight gain (WG) in animals [34]. Moreover, previous studies have demonstrated that the use of antibiotics in humans, especially in infants and children, is also associated with WG [21]. The mechanism by which antibacterial agents improve growth performance is not well known, but several hypotheses have been proposed [16]: (i) nutrients are more efficiently absorbed because of a thinner small-intestinal epithelium; (ii) nutrients are spared because competing microorganisms are reduced; (iii) microorganisms responsible for subclinical infections are reduced or eliminated; (iv) production of growth-depressing toxins or metabolites by intestinal microflora is reduced; or (v) modifications of bacterial enzyme activity improve food efficiency. Thus, there is increasing evidence about the role of the gut's microorganisms on the energy conversion of nutriments [8] and their implications on obesity. This has been supported by reports involving mouse models with a genetic tendency for obesity [9], [13], [15]. A recent 16S rRNA sequencing-based investigation showed that the *Firmicutes*/*Bacteroidetes* ratio differed in obese and lean mammals, mainly due to an increased *Firmicutes* proportion in obese mammals [15]. *Lactobacillus* sp, one of these bacterial communities with probiotic activity, has an impact on food conversion and a potential effect on WG, as demonstrated in farm animals [26], [27], [28]. *Lactobacillus* sp treatment results in higher metabolic activity, lower levels of non-esterified fatty acids, triglycerides, urea, and an increase in the levels of alkaline phosphatase and creatine kinase [35]. Vendt *et al.* treated 120 healthy infants (up to two months), with either regular formula, or formula supplemented with 10^7^ CFU/g *L. rhamnosus* strain GG. After six months, the 51 children that received the *Lactobacillus*-supplemented formula gained weight more rapidly and displayed significantly greater body length and weight than those receiving the regular formula [29].

These well known data concerning the impact of avoparcin and *Lactobacillus* sp on WG in animals and humans may explain the results found with vancomycin in the present study. Indeed, vancomycin is an analogue of avoparcin, and *Lactobacillus* sp are known to be resistant to glycopeptides [24], [25]. Thus, we can speculate that the weight gain was induced by the growth promoter effect of *Lactobacillus* sp in patients who had been treated by vancomycin. Moreover, a previous study in rats demonstrated that amoxicillin induced alterations in microbial populations that included depletion of the *Lactobacillus* sp [36]. These results are in accordance with the absence of significant weight gain observed in our patients treated with amoxicillin.

The empirical data from agriculture and experimental data in laboratory animals show that manipulating gut microbiota by antibiotic administration or by colonization with selected bacteria results in significant WG. We speculate that some antibiotics and probiotics may have the same effect in humans. A recent study shows that one cause of an abrupt shift in intestinal microbiota in babies is antibiotic treatment [10]. Thus, a possible link between increased antibiotic use and obesity has been recently proposed [22], [37], and our preliminary results add credence to this hypothesis.

Moreover, our stratified analysis showed the impact of three factors that could influence weight changes during antibiotic treatment: cardiac surgery, age, and gender. First, cardiac surgery could have limited the WG in some patients because of a longer hospitalization, especially in intensive care units where the calorific intake is not optimal. Second, the fact that older patients had a greater WG may be explained by a higher proportion of treatment without surgery in this subgroup. Finally, the trend towards a greater WG in males could be either related to the fact that females pay more attention to their weight changes than males in our country [38] or to the relatively small sample size of females in the present work.

It should be noted that the present study has several limitations, including the small sample size and the absence of a systematic, prospectively collected questionnaire of lifestyle. However, with our eligibility criteria, the profile of patients in the two groups was similar because it represents the relatively homogenous sample of those in whom IE was suspected. Moreover, the history of cancer that was more frequently noticed in the Antibiotic group emphasizes the results on weight gain observed in that group. Finally, our hypothesis about the colonization of the gut by *Lactobacillus* sp in patients treated with vancomycin is at this time only speculative and needs to be confirmed. We are currently testing this hypothesis.

## Conclusion

Significant and persistent WG can occur after treatment with high doses of vancomycin in patients with IE. The risk of obesity is particularly high in vancomycin-treated male patients older than 65 who did not undergo cardiac surgery. If these results are confirmed by other studies, it will be important to prevent major WG, possible obesity, and all of their associated consequences after a long treatment with antibiotics. Hence, we suggest nutritional programs and weight follow-up in patients undergoing such treatment, especially those with a history of heart disease. Finally, we hypothesize that the growth promoter effect of vancomycin might be explained by the selection of *Lactobacillus* sp in the gut microbial flora.
